# Hospitalizations realted to herpes zoster infection in the Canary Islands, Spain (2005-2014)

**DOI:** 10.1186/s12879-017-2688-y

**Published:** 2017-08-24

**Authors:** Amós García-Rojas, Ruth Gil-Prieto, Domingo Ángel Núñez-Gallo, Petra Matute-Cruz, Angel Gil-de-Miguel

**Affiliations:** 1 Epidemiology and Prevention Service, Public Health General Direction, Las Palmas de Gran Canaria, Spain; 2 Area of Preventive Medicine & Public Health, Rey Juan Carlos University, Avda. Atenas s/n, 28922 Madrid, Spain

**Keywords:** Hospitalizations, Herpes zoster, Epidemiology

## Abstract

**Background:**

Herpes zoster is an important problem of public health especially among the elderly in Spain.

**Methods:**

A population-based retrospective epidemiological study to estimate the burden of herpes zoster requiring hospitalization in the Canary Islands, Spain was conducted by using data from the national surveillance system for hospital data, Conjunto Mínimo Básico de Datos. Records of all patients admitted to hospital with a diagnosis of herpes zoster in any position and cases of primary diagnosis (ICD-9-MC codes 053.0–053.9) during a 10-year period (2005–2014), were selected.

**Results:**

A total of 1088 hospitalizations with a primary or secondary diagnosis of herpes zoster were identified during the study period. Annually there were 6.99 hospitalizations by herpes zoster per 100,000 population. It increases with age reaching a maximum in persons ≥85 years of age (43.98 admissions per 100,000). Average length of hospitalization was 16 days and 73 patients died, with a case-fatality rate of 4.03%. In 22% of the cases hospitalized, herpes zoster was the primary diagnosis.

**Conclusion:**

The hospitalization burden of herpes zoster in adults in the Canary Islands was still important during the last decade and justify the implementation of preventive measures, like vaccination in the elderly or other high risk groups to reduce the most severe cases of the disease.

**Electronic supplementary material:**

The online version of this article (doi:10.1186/s12879-017-2688-y) contains supplementary material, which is available to authorized users.

## Background

It is estimated that the 15% of the global population will suffer from least one episode of herpes zoster during their lifetime [1]. With an increase in age, loss of cell-mediated immunity and immunosenescence, complications of herpes-zoster may occur [2, 3], being post-herpetic neuralgia the most frequent and limititing [4, 5], causing important pain to 15–40% of the patients majorly impacting quality of life [3, 6].

Currently in Spain, there are two licensed varicella vaccines indicated for active immunization for the prevention of varicella in susceptible individuals 12 months of age or older. In addition, one licensed herpes zoster vaccine indicated for prevention of herpes zoster in healthy immunocompetent individuals 60 years of age and older susceptible of having herpes zoster and post-herpetic neuralgia caused by varicella zoster virus (VZV) [7]. Herpes zoster vaccine is not funded as part of a government immunisation program. Starting in 2016 varicella vaccines were included as part of the government funded immunisation program at 15 months and 3 years of age in the Canary Islands. It is essential to study the burden of herpes zoster and its epidemiology in order to correctly evaluate the efficacy, effectiveness and impact of different preventive strategies, like the use of varicella vaccine in routine vaccination schedules in children, susceptible adolescents or herpes zoster vaccine in the elderly or adults in high risk groups. The use of hospitalization data bases for studying the epidemiology of different infectious diseases is widespread as they are not subject to mayor under-diagnosis and give a good spectrum of the most severe cases of the disease. [8].

The objective of this study was to report the hospitalization burden related to herpes zoster infection in the Canary Islands from 2005 to 2014.

## Methods

A retrospective epidemiological study was performed by using hospitalization discharge registers from the national information system for hospital data (CMBD, Conjunto Mínimo Básico de Datos), from the Spanish Ministry of Health. All the registers for the Canary Islands, with an avarage population of 2,033,201, reported from January 1st, 2005 through December 31st, 2014 were selected. The national information system for hospital data (Conjunto Mínimo Básico de Datos; CMBD) includes an estimated 98% of discharges in public hospitals, covering 99.5% of the Spanish population. [9–11].

The 9th International Classification of Diseases ICD-9-CM (CIE-9-MC) [12] codes for herpes zoster (053.0, 053.1, 053.10,053.11, 053.12, 053.13, 053.19, 053.2, 053.20, 053.21, 053.22,053.29, 053.7, 053.71, 053.79, 053.8, 053.9) were selected. All herpes zoster related hospitalizations in a) any diagnostic position and b) first diagnostic position were analyzed..

This data base has been extensively used in epidemiological studies evaluating the burden of disease and changes in epidemiology of different diseases like varicella, respiratory syncytial virus or anaphylaxis, among others [13–15].

For each case, information on age, sex, outcome (recovery, death…) and length of stay was obtained.

### Statistics

The unit of analyses was the hospital discharge. The annual hospitalization rate (annual number of hospital admissions per 100,000 population), average length of hospital stay (ALOS), mortality rate (annual number of deaths at hospital per 100,000 population) and case-fatality rate (annual number of deaths at hospital/annual number of hospital admissions; %) were calculated. Data from the age- specific annual municipal population registries (corrected by the CMBD coverage) were used as the denominator. It was assumed that the distribution by age of the population covered by this data base was equal to the distribution of the general population of the Canary Islands.

All analyses were stratified by age group: < 50 years, 50–54 years, 55–59 years, 60–64 years, 65–69 years, 70–74 years, 75–79 years, 80–84 years and ≥85 years. We also reported the crude and age-standardized (EU27 + EFTA 2013 standard population) total hospitalization, total mortality, and total case fatality rates. ANOVA or Kruskal Wallis tests were used to evaluate differences in means and distributions. Poisson regression was used to assess differences in the hospitalization rate over the study period.

In all tests the significance level used was *p* < 0.05. Statistical analyses were performed using the IBM Statistical Package for Social Sciences (IBM SPSS/PASW for windows, version 21.0, Chicago, IL, USA).

The patient information was anonymized and de-identified prior to the analysis. Local ethics committee (Comité de Ética de la Investigación de la Universidad Rey Juan Carlos) ruled that no formal ethics approval was required in this particular case.

## Results

A total of 1088 hospital discharges related to herpes zoster infection were reported during the 10-year study period in the Canary Islands, with an annual crude hospitalization rate of 5.35 (95%-CI 5.03–5.67) cases per 100,000 inhabitants (age standardized hospitalization rate = 6.99, 95%-CI = 6.57–7.43). The average length of stay was 15.82 (SD 18.84) days and their mean age was 63.69 (SD 19.89) years old. Sixty-eight percent of the hospitalizations occurred in patients 60 years of age or older and 35% in patients aged more than 75. There were a total of 73 deaths among the patients hospitalized with herpes zoster during the study period. The mortality rate was 0.36 (95%-CI = 0.28–044) deaths per 100,000 inhabitants (age standardized: 0.51, 95%-CI = 0.40–0.65) and the case fatality rate was 6.71% (95%-CI = 5.22–8.20, age standardized 4.03%, 95%-CI = 2.78–5.86.

Analysis restricting to 263 herpes zoster cases registered in the first diagnostic position only were reported in Additional file 1: Table S1 yielding a total hospitalization rate of 1.29 (95%-CI = 1.14–1.45) hospitalizations in the first diagnostic position per 100,000 inhabitants of the Canary Islands (1.54, 95%-CI = 1.35–1.74 per 100,000 inhabitants after ager standardization). Of these hospitalizations, 47 (18%) were related to herpes zoster with meningitis (053.0), 56 (21%) to herpes zoster with other nervous system complications (053.1), 39 (15%) to herpes zoster with ophthalmic complications (053.2), 36 (14%) to herpes zoster with other specified complications (053.7), 1 (0%) to herpes zoster with unspecified complications (053.8), and 84 (32%) to herpes zoster without complications (053.9).

When analyzing rates per group of age, both hospitalization and mortality rates increased in a statistically significant way with age, reaching the maximum values in the elderly (>84 years old) with 43.98 cases per 100,000 inhabitants (95%-CI = 36.08–51.88) and 5.17 deaths per 100,000 inhabitants (95%-CI: 2.46–7.89), respectively (Table 1). Case-fatality rates also increased with age, but statistical significance was not reached. (Table 1). The highest case-fatality rate was found in the 75–79 group of age with 12.21% (CI 95% 6.61–17.82).

**Table 1:** Hospitalization rate, mortality rate and case-fatality rate related to herpes zoster infection by group of age in the Canary Islands, Spain (2005–2014)

Group of age (years)	N	Cases	Hospitalization rate (cases per 100.000 /CI95%)	Mortality rate (deaths per 100.000 /CI95%)	Case-fatality rate (% /CI95%)	Average length of hospital stay (days/SD)
<50	1,420,480	241	1.70	0.04	2.08	12.73
			1.48–1.91	0.004–0.07	0.28–3.87	13.69
50–54	132,854	49	3.69	0.23	6.12	17.31
			2.66–4.72	0.00–0.48	0,12.84	13.69
55–59	112,155	61	5.44	0.27	4.92	15.02
			4.07–6.80	0–0.57	0–10.35	11.86
60–64	97,906	109	11.13	0.82	7.34	18.01
			9.04–13.22	0.25–1.38	2.44–12.26	21.69
64–69	82,777	123	14.86	1.09	7.32	20.95
			12.23–17.49	0.37–1.80	2.72–11.92	35.07
70–74	70,038	128	18.28	0.71	3.91	15.36
			15.11–21.44	0.09–1.34	0.55–7.26	14.88
75–79	55,721	131	23.51	2.87	12.21	17.04
			19.49–27.54	1.46–4.28	6.61–17.82	17.01
80–84	35,214	127	36.07	2.84	7.87	14.94
			29.79–42.34	1.08–4.60	3.19–12.56	13.57
>84	26,056	119	43.98	5.17	11.77	14.70
			36.08–51.88	2.46–7.89	5.98–17.55	13.50
Total		1088	5.35	0.36	6.71	15.82
			5.03–5.67	0.28–0.44	5.22–8.20	18.84
Total (age-standardized)^a^			6.99	0.51	4.03	
			6.57–7.43	0.40–0.65	2.78–5.86	

^a^standardized to the EU-27 + EFTA standard population from http://ec.europa.eu/eurostat/en/web/products-manuals-and-guidelines/-/KS-RA-13-028

The hospitalization rates did not suffer important variations during the study period, except for the oldest group of age, 85+ where there was a significant increase in the hospitalization rates. (*p* < 0.05; Table 2).

**Table 2:** Hospitalization rate (cases per 100.000 /CI95%) related to herpes zoster infection by group of age in the Canary Islands, Spain (2005–2014)

		2005		2006		2007		2008		2009		2010		2011		2012		2013		2014
Group of age (years)	N	Rate	N	Rate	N	Rate	N	Rate	N	Rate	N	Rate	N	Rate	N	Rate	N	Rate	N	Rate
<50	27	1.93	21	1.49	18	1.27	16	1.11	26	1.79	20	1.38	32	2.23	18	1.27	31	2.22	32	2.33
		1.20–2.65		0.85–2.13		0.68–1.85		0.56–1.65		1.10–2.48		0.78–1.99		1.46–3.00		0.69–1.86		1.44–3.00		1.52–3.13
50–54	8	7.18	2	1.73	5	4.16	5	3.98	6	4.59	5	3.70	3	2.13	5	3.46	4	2.67	6	3.88
		2.21–12.16		0–4.13		0.51–7.81		0.49–7.47		0.92–8.26		0.46–6.94		0–4.54		0.43–6.50		0.05–5.28		0.78–6.98
54–59	5	5.10	7	6.99	5	4.83	2	1.86	8	7.19	10	8.73	2	1.70	5	4.16	8	6.49	9	7.18
		0.63–9,58		1.81–12.16		0.60–9.06		0–4.43		2.21–12.17		3.32–14.14/		0–4.06		0.51–7.08		1.99–11.00		2.59–11.87
60–64	7	8.19	7	7.87	13	13.98	9	9.39	11	11.14	12	11.92	16	15.69	9	8.72	12	11.40	13	12.28
		2.12–14.26		2.04–13.70		6.38–21.58		3.26–15.53		4.56–17.72		5.18–18.67		8.00–23.37		3.02–14.42		4.95–17,84		5.60–18.95
64–69	5	7.05	13	17.89	10	13.79	10	13.05	14	17.28	12	13.80	19	21.29	15	16.29	12	12.91	13	14.02
		0.97–13.21		8.17–27.61		5.24–22.34		4.96–21.14		8.23–26.34		5.99–21.60		11.72–30.86		8.05–24.53		5.61–22.79		6.40–21.64
70–74	11	16.48	10	14.47	11	15.42	14	19.57	16	22.64	15	21.85	12	17.20	13	18.76	10	14.07	16	22.16
		6.74–26.22		5.50–23.44		6.31–24.53		9.32–29.82		11.55–33.73		10.79–32.91		7.47–26.93		8.57–28.96		5.35–22.79		11.30–33.02
75–79	5	11.18	4	8.48	19	38.32	12	22.50	15	26.61	16	26.93	13	21.10	19	29.96	20	32.10	8	13.50
		1.38–20.98		0.17–16.79		21.09–55.54		9.77–35.23		13.15–40.08		13.74–40.12		9.63–32.57		16.49–43.42		18.03–46.17		4.15–22.86
80–84	11	39.23	4	13.72	8	26.42	10	31.44	11	32.72	22	61.80	11	29.13	22	55.09	9	21.23	19	43.63
		16.05–62.41		0.28–27.17		8.12–44.73		11.96–50.92		13.39–52.05		35.98–87.61		11.92–46.33		32.08–78.10		7.36–35.10		24.02–63.25
>84	5	22.08	7	29.94	7	30.17	12	49.04	9	34.89	19	68.57	13	44.55	11	36.19	21	66.89	15	46.32
		2.73–41.42		7.77–52.12		7.82–52.52		21.30–76.77		12.10–57.68		37.75–99.39		20.34–68.76		14.81–57.58		38.29–95.49		22.88–69.75
Total	84	4.36	75	3.84	96	4.84	90	4.42	116	5.63	131	6.31	121	5.81	117	5.64	127	6.12	131	6.35
		3.42–5.29		2.97–4.70		3.87–5.80		3.51–5.34		4.60–6.65		5.23–7.39		4.77–6.84		4.62–6.66		5.05–7.18		5.26–7.44
Total (age-standardized)^a^		5.69		5.09		6.69		6.25		7.39		8.57		7.28		7.13		7.55		7.56
		4.49–7.16		3.97–6.49		5.39–8.27		4.99–7.76		6.07–8.94		7.14–10.24		6.01–8.76		5.88–8.59		6.27–9.03		6.30–9.02

^a^standardized to the EU-27 + EFTA standard population from http://ec.europa.eu/eurostat/en/web/products-manuals-and-guidelines/-/KS-RA-13-028

## Discussion

This is the first study to the date assessing the hospital burden of herpes zoster in the Canary Islands and its trend in the last decade. Obtained crude hospitalization rates during the period of 5.35 (6.99 when age-standardized) cases per 100,000 inhabitants are in line with the regional results (6.1 cases per 100,000 in the Canary Islands) published in a national study with data from 1998 to 2004 for adults older than 30 years old [15]. In that study it was seen that the Canary Islands had the lowest hospitalization rate and the longest average length of stay at hospital of all the regions of Spain, suggesting that only very severe cases were being admitted. In the current study, a slightly shorter length of hospitalization of 16 days (vs. 20 days in 1998–2004) was found.

When comparing hospitalization rates in the Canary Islands with Spanish national figures from 2005 to 2010, our results are significantly lower than the reported national rate of 10.33 hospitalizations per 100,000 inhabitants [16]. Regional differences of the epidemiology of herpes zoster regarding Spanish national data may be explained by the special characteristics of age distribution of the population of the Canary Islands, with an important number of young immigrants from African countries and of old immigrants from Northern European countries. Another contributing factor may the special geographic characteristics of the Canary Islands located in a tropical area where the seroprevalence of varicella may be lower.

The dramatic increase of hospitalization rates with age, especially from 60 to 70 years of age, follows the pattern observed in other occidental countries, such as the United Kindom [17, 18].

The main result of this study is to show the important hospital burden of herpes zoster, especially in the elderly. The increase of the hospitalization rate in the oldest group of age is very interesting. But this increase has been previously explained as a result of the increase in mean age of the group of 85+ years old due to population ageing and the increasing life expectancy and was shown not to be related with the more or less extensive use of the varicella vaccine in young children [16]. Supporting this theory is the 3.2% annual increase of hospitalization rate due to any cause that was reported in Spain over the study period.

Although the use of population data bases to study infectious diseases epidemiology has been extensively proven worldwide, it has some limitations that should be considered. First, considering herpes zoster diagnosis code in any diagnosis position could overestimate the real hospital burden related to herpes zoster infection, as patients could be admitted due to other causes. For the purpose of the present project, we considered primarily considered the presence of herpes zoster in any diagnostic position because having herpes zoster infection could worsen or lengthen the hospitalization by any other cause thus contributing to the burden of herpes zoster. For completeness and to get a better perspective on the incidence of herpes zoster hospitalizations and its complications, we reported results when using only the first diagnostic position in the text and the appendix. It should be mentioned that only hospitalized cases are included in this study and hence only the most severe spectrum of the disease. Our results should therefore not be interpreted as disease incidence as the outpatient setting is not included. Although CMBD does not necessary include microbiological diagnosis, the clinical diagnosis of herpes zoster and post herpetic neuralgia is very specific and some studies including chart reviews have shown a positive predictive value between 89–96% [19].

## Conclusion

The hospitalization burden of herpes zoster in adults in the Canary Islands was still important during the last decade and justify the implementation of preventive measures, like vaccination in the elderly or high risk groups, to at least reduce the most severe cases of the disease.

## Additional file

Additional file 1: Table S1 Hospitalization rate, mortality rate and case-fatality rate related to herpes zoster infection by group of age in the Canary Islands, Spain (2005–2014) – Principal Diagnosis only. (DOCX 13 kb)

## Abbreviations

ALOS: Average length of hospital stay
CI: Confidence interval
CMBD: Conjunto Mínimo Básico de Datos
ICD: International Classification of Diseases
VZV: Varicella zoster virus
