# Supplementary material for: Hospitalizations realted to herpes zoster infection in the Canary Islands, Spain (2005-2014)
Source: BMC Infect Dis. 2017 Aug 24;17:586. doi: 10.1186/s12879-017-2688-y (PMC5571588; doi:10.1186/s12879-017-2688-y)
Supplement: Additional file 1: — Table S1 Hospitalization rate, mortality rate and case-fatality rate related to herpes zoster infection by group of age in the Canary Islands, Spain (2005–2014) – Principal Diagnosis only. (DOCX 13 kb) [file 12879_2017_2688_MOESM1_ESM.docx]

Table S1 Hospitalization rate, mortality rate and case-fatality rate related to herpes zoster infection by group of age in the Canary Islands, Spain (2005-2014) – Principal Diagnosis only.

| **Group of age (years)** | **N** | **Cases** | **Hospitalization rate**  **(cases per 100.000 /CI95%)** | **Mortality rate**  **(deaths per 100.000 /CI95%)** | **Case-fatality rate**  **(% /CI95%)** | **Average length of hospital stay**  **(days/SD)** |
| --- | --- | --- | --- | --- | --- | --- |
| **<50** | 1,420,480 | 98 | 0.69  0.55-0.83 | 0.01  0-0.21 | 1.02  0-3.01 | 8.28  5.49 |
| **50-54** | 132,854 | 14 | 1.05  0.50-1.61 | 0.08  0-0.22 | 7.14  0-20.63 | 10.43  10.01 |
| **55-59** | 112,155 | 16 | 1.43  0.73-2.13 | 0 | 0 | 10.38  3.14 |
| **60-64** | 97,906 | 28 | 2.86  1.80-3.92 | 0.10  0-0.30 | 3.57  0-10.45 | 9.43  4.92 |
| **64-69** | 82,777 | 21 | 2.54  1..45-3.62 | 0 | 0 | 25.52  40.82 |
| **70-74** | 70,038 | 30 | 4.28  2.75-5.82 | 0.14  0-0.42 | 3.33  0-9.76 | 13.13  10.61 |
| **75-79** | 55,721 | 27 | 4.85  3.02-6.67 | 0.36  0,0.86 | 7.41  0-17.29 | 15.56  20.74 |
| **80-84** | 35,214 | 18 | 5.11  2.75-7.47 | 0 | 0 | 15.56  15.70 |
| **>84** | 26,056 | 11 | 4.07  1.66-6.47 | 0 | 0 | 11.27  8.53 |
| ***Total*** |  | 263 | 1.29  1.14-1.45 | 0.03  0.01-0.05 | 2.28  0.48-4.09 | 11.94  15.61 |
| ***Total (age-standardized)**** |  |  | 1.54  1.35-1.74 | 0.04  0.01-0.09 | 1.80  0.52-4.92 |  |
